# Anthropogenic and Ecological Drivers of Amphibian Disease (Ranavirosis)

**DOI:** 10.1371/journal.pone.0127037

**Published:** 2015-06-03

**Authors:** Alexandra C. North, David J. Hodgson, Stephen J. Price, Amber G. F. Griffiths

**Affiliations:** 1 Environment and Sustainability Institute, University of Exeter, Penryn Campus, Penryn, Cornwall, United Kingdom; 2 Centre for Ecology and Conservation, University of Exeter, Penryn Campus, Penryn, Cornwall, United Kingdom; 3 UCL Genetics Institute, Gower Street, London, United Kingdom; University of South Dakota, UNITED STATES

## Abstract

Ranaviruses are causing mass amphibian die-offs in North America, Europe and Asia, and have been implicated in the decline of common frog (*Rana temporaria*) populations in the UK. Despite this, we have very little understanding of the environmental drivers of disease occurrence and prevalence. Using a long term (1992-2000) dataset of public reports of amphibian mortalities, we assess a set of potential predictors of the occurrence and prevalence of *Ranavirus*-consistent common frog mortality events in Britain. We reveal the influence of biotic and abiotic drivers of this disease, with many of these abiotic characteristics being anthropogenic. Whilst controlling for the geographic distribution of mortality events, disease prevalence increases with increasing frog population density, presence of fish and wild newts, increasing pond depth and the use of garden chemicals. The presence of an alternative host reduces prevalence, potentially indicating a dilution effect. Ranavirosis occurrence is associated with the presence of toads, an urban setting and the use of fish care products, providing insight into the causes of emergence of disease. Links between occurrence, prevalence, pond characteristics and garden management practices provides useful management implications for reducing the impacts of *Ranavirus* in the wild.

## Introduction

Amphibians are the most endangered taxonomic group on the planet, with one third of species currently holding a threatened status (IUCN categories Vulnerable, Endangered or Critically Endangered; [1], [2]). Emerging diseases are one main driver of these amphibian declines [3], alongside a range of other threats including over-exploitation, habitat loss and climate change [4]. Ranaviruses impact amphibians worldwide [5] and are causing notable die-offs in North America, Europe and Asia [5–11]. The pathogen has been implicated in population declines of several European species [10] including declines of over 80% in UK common frogs (*Rana temporaria*) [9] and models suggest that *Ranavirus* has the potential to cause local extinction in wood frog (*Lithobates sylvaticus*) populations [12].

Ranaviruses are large, double-stranded DNA viruses belonging to the family *Iridoviridae* [13] that infect amphibians, fish and reptiles [14], [15]. They can cause systemic disease (ranavirosis), resulting in cell death [13] and overt signs—ulcerations, haemorrhaging, muscle necrosis or lip erythema [16]—which may depend on the stage of disease progression [17]. In the wild, *Ranavirus* induced mortality rates can be in excess of 90%, with year on year re-occurrence of die-offs in the same amphibian populations [7] and observed die-offs as large as 200 individuals [9]. Susceptibility to *Ranavirus* does however differ between host developmental stages [18], populations [19], and species [15], [20], and is influenced by a range of biotic and abiotic characteristics, including differences in habitat [20] and temperature [21]. Differences in susceptibility may also relate to the virus genotype [22], though only a single species of *Ranavirus* is thought to be present in the UK [14]. Incidence of mortality events can show seasonal variation, peaking in summer months in the UK [23] in contrast to North America where incidence is highest in autumn and winter [24].

Emerging pathogens are classed as novel if, for example, they have been introduced to new regions by humans, and endemic if they were already present in a region but have moved into a new host or increased in pathogenicity [25]. The distinction is important since the different histories will likely warrant different management approaches. *Ranavirus* spread has been strongly linked to human activity, including international trade [14], [26]—[28], the use of infected salamanders as fishing bait [29] and industrial and agricultural activities [30]. Modelling the emergence of ranavirosis in the UK reveals human population density as an important predictor of spread [17], though it is challenging to determine which aspects of human activity are responsible.

Commonly used anthropogenic chemicals such as pesticides are known to cause immunosuppression in amphibians [31]. The herbicide atrazine for example reduces leukocyte production and increases host susceptibility to *Ranavirus* [32], and the insecticide chlorpyrifos increases *Ranavirus* infection rates in the tiger salamander (*Ambystoma tigrinum*) [33]. Carbyl insecticide has also been shown to decrease host survival when in combination with *Ranavirus* and predator cues [34]. Few studies have however looked at the relationship between chemical use and *Ranavirus* infection in free living populations, and those that have, focus on North American species and correlates such as distance to agricultural or industrial activity [30] or other abiotic contaminants such as aluminium [35] and water ammonia levels [36].

Biotic characteristics such as the presence of alternative host species have also been shown to influence the incidence and impact of *Ranavirus*. Identical ranaviruses have been isolated from wild sympatric amphibians and fish, suggesting one could be a reservoir for the other [37]. Ranaviruses have also been isolated from a range of economically important fish [38], and it has been suggested that *Ranavirus* may have entered the UK through goldfish and bullfrog imports from North America [14], [26]. In the UK *Ranavirus* infection is also associated with common toads (*Bufo bufo)*, with known susceptibility of common frogs to virus isolated from a toad [39]. Common toads were less susceptible than common frogs to experimental challenge with a number of different *Ranavirus* isolates; toad tadpoles showed lower prevalence of infection and fewer signs of disease [40]. Smooth newts (*Lissotriton vulgaris*) have also tested positive for *Ranavirus* [41], and in Europe mass mortalities of palmate newts (*Lissotriton helveticus*) as well as alpine newts (*Mesotriton alpestris*), an introduced species in the UK [42], have been associated with *Ranavirus* infection [8].

The availability of alternative hosts within a multi-host system affects transmission dynamics [43]. Inter-specific transmission can mean counts of all available hosts contribute to total host density and promote pathogen persistence when transmission is density dependent. In some cases an amplification effect may be seen, whereby each species contributes to the pool of susceptible hosts, so that pathogen abundance may be higher than in the presence of just one host species alone [43]. Alternatively, if additional hosts are of lower quality, their presence may decrease the pathogen’s ability to persist [44], [45]. The mechanisms behind this decrease are likely to vary according to transmission dynamics and the complexity of the system in question [46], [47]. Generally, the presence of poor quality hosts may result in fewer new infections compared to in the presence of the primary host alone, as a result of decreased encounter rates between primary hosts [47], reduced downstream transmission or reduced propagule production [46]. This is referred to as a dilution effect [43], [45], [47] and has been reported in North American anurans infected with the fungal pathogen *Batrachochytrium dendrobatidis* [48].

The amphibian life history stage most susceptible to *Ranavirus* infection varies geographically: adults appear worst affected in UK common frogs [49] compared to larvae and recent metamorphs in North American anurans [7]. In the UK, vertical transmission seems unlikely, as *Ranavirus* levels in early life history stages are negligible [49] and mathematical models suggest adult to adult transmission could be sufficient to allow persistence [50]. Larger *Ranavirus*-consistent die-offs were associated with larger populations in common frogs [9], which could highlight a role for density in transmission. Density dependent transmission would result in natural regulation of disease levels, due to differing transmission efficiencies at different host densities, enabling host population recovery at low contact rates. However, as *Ranavirus* is a multi-host pathogen [7], [15] and can persist in sediment and water [51–53] it seems likely that the virus could be maintained at low host densities too. A better understanding of factors associated with outbreaks of ranavirosis could provide useful insights into the transmission dynamics at play.

In this study we analysed a long term dataset of common frog mortalities from across Britain to identify characteristics associated with ranavirosis occurrence and prevalence. Ranavirosis occurrence was defined as the presence or absence of *Ranavirus*-consistent deaths at a site, and prevalence was defined as the proportion of the estimated total frog population killed in the mortality event. Only one species from the genus *Ranavirus* is known to be present in the UK (Frog Virus 3) and so here the species is assumed to be consistent across mortality events. Possible predictors focused on (i) biotic characteristics including the presence of alternate hosts (toads, newts and ornamental fish) and common frog population density, and (ii) abiotic characteristics including pond depth, level of urbanisation, pond management (use of fish care products) and the use of garden chemicals (pesticides and herbicides) (See S1 Table for *a priori* justification of the choice of predictors).

## Methods

### Study System

Common frogs are one of seven native amphibian species found in the UK [54]. They are thought to be largely philopatric, with relatively short maximum dispersal distances recorded [55], [56]. In the UK, it is the adult life stage of common frogs that is impacted by *Ranavirus* [23], [49]. Much of what we know about *Ranavirus* transmission is based on infection trials in a laboratory setting [39], [57]. Experimental trials found both the route of exposure and the source of *Ranavirus* to influence disease development and mortality rates in common frogs, suggesting transmission could occur through direct contact and via exposure to infected water [57].

### Frog Mortality Project Database

In the UK, common frogs (*Rana temporaria*) are frequently found in private artificial garden ponds, often in urban and sub-urban settings. This provides a unique opportunity for members of the public to monitor wild populations. Since 1992, UK pond owners have submitted reports of common frog mass mortality events to the Frog Mortality Project (FMP), administered by Froglife (UK registered charity no. 1093372). Reports were originally encouraged via nationwide media appeals to the general public, conservation organisations and animal welfare groups. Mortality reports were initially filed by paper questionnaires and followed up by phone calls to ensure the accuracy of the information received. Later reports were filed via a mix of paper and electronic questionnaires. All reports were subsequently consolidated in a digital database which is analysed here. Species identification is relatively straightforward, as the only common, native UK species are common frogs, common toads, palmate, smooth (*Lissotriton vulgaris*) and great crested newts (*Triturus cristatus*). Pool frogs (*Pelophylax lessonae*) and natterjack toads (*Bufo calamita*) may also be present, but these species are rare with restricted ranges [54], [58].

Here we have analysed mortality reports of diseased and non-diseased amphibians submitted by the public between 1992 and 2000 (Maximum complete reports analysed here, n = 2,219, although some analyses use subsets of the dataset). Each report lists the number of deaths, any signs of disease or injury observed, an estimated healthy population size and details about the pond and garden management. Total frog population size estimates were validated via correlation analyses between additional independent measures of population size recorded within the database (For details see S1 File). Due to the citizen science nature of the dataset, additional explanatory variables could not be formally validated. Anecdotal evidence from authors working alongside pond owners who have contributed to this database suggests reporters are reliable and conscientious. Whilst the lack of formal validation means our results should be interpreted with caution, large sample sizes recommend confidence in the patterns detected. A set of criteria for filtering database records has been applied previously to identify *Ranavirus-*consistent mortality events within the FMP database and established this method as a reliable predictor of *Ranavirus* infection [9]. Records identified as *Ranavirus*-consistent by these criteria were validated through molecular screening of frogs for infection and gross examination for signs of past infection. The disease status of all carcasses screened matched the disease status classified using the filtering criteria [9]. These criteria were (i) mortality event occurring between the warmer months of May-September due to the peak occurrence of *Ranavirus* disease in warmer months and to exclude winterkill related mortalities which is thought to be the other main cause of mass mortalities in the UK [23], and (ii) pathognomonic signs of ulcerations or highly diagnostic systemic haemorrhaging [9] (Criteria 1, *Ranavirus*-consistent positive events: n = 702). These overt signs are not associated with any other pathogen in the UK, and interactions with winter mortality are highly unlikely since winter mortality is strongly associated with bloated and pale bodies, neither of which are signs associated with *Ranavirus* mortality [23]. To ensure the robustness of any conclusions made from these criteria, analyses were also conducted using a second previously defined set of criteria [17]. This second set of criteria removes the requirement for events to occur during summer months and defines a *Ranavirus*-consistent mortality event as (i) signs of systemic haemorrhaging, ulcerations or limb necrosis, alongside (ii) at least five deaths in the mortality event (Criteria 2, *Ranavirus*-consistent positive events: n = 740).

The inclusion of non-summer mortality events in criteria 2 was considered to be the largest difference between criteria. To determine the cause of any differences in the results between criteria, criteria 2 [17] was subsequently restricted to exclude mortalities outside of May-September as per criteria 1 [9] (*Ranavirus*-consistent events: n = 653).

### Statistical analysis

A multi-model inference approach was adopted with all possible combinations of main effects being ranked according to their (Q)/AIC (Quasi/Akaike Information Criterion). The AIC provides an estimate of the Kullback-Leibler distance and can be used to select the best fitting model or set of models [59]. Model averaging was conducted across all models with *Δ*(Q)/AIC < 6 [60] to account for model selection uncertainty and to determine the effect size and direction of variables influencing ranavirosis occurrence and prevalence [61]. All analyses were conducted in R [62] using packages mgcv for generalized additive models [63] and MuMIN for model ranking and averaging [64]. To control for duplicate records through time, the first event from each postcode was included and subsequent duplicate records removed.

#### a) Factors affecting ranavirosis occurrence

Ranavirosis occurrence was defined as the presence or absence of *Ranavirus*-consistent deaths at a site and is a binary response term in this global model (presence/absence). Nine explanatory variables were included (See S1 Table for *a priori* justification). Biotic predictors included: fish (91% of ponds within the database that specified fish had goldfish varieties, 24% had koi, 19% had orfe and 12% had tench), newt (species unspecified) and toad presence (species unspecified but assumed to be largely common toads due to the highly restricted range and specialised habitat requirements of natterjack toads [54], [65]), frog population density (total population size divided by maximum total pond volume). Abiotic predictors included: average pond depth, level of urbanisation (urban/ rural) and whether chemicals such as herbicides, slug pellets or fish care products were used in the garden (Criteria 1: n = 2,113, Criteria 2: n = 2,219, Criteria 2 excluding mortalities outside of May-September: n = 2,160). Missing data and uncertain species identification meant we addressed fish as a class and not at the level of species.

#### b) Factors affecting ranavirosis prevalence

Ranavirosis prevalence was defined as the proportion of the estimated total frog population killed in the mortality event. Analysis of prevalence used the subset of the data containing *Ranavirus*-consistent events only (Criteria 1: n = 702, Criteria 2: n = 740, Criteria 2 excluding mortalities outside of May-September: n = 653). Estimated total frog population size was used to compute a log-odds ratio of ranavirosis-caused frog deaths compared to the total population, analysed with a binomial error structure. All explanatory variables used in the occurrence analysis were included.

Spatial non-independence of residuals was determined by the significance of a spatial smoothing term in generalized additive models and by confirming improved model fits of spatial versus non-spatial models. Generalized additive models with binomial error structures were subsequently used with a smoothing term to account for the geographic pattern of disease events. Covariates were modelled as parametric terms and the northings and eastings of each mortality event were included as a non-parametric thin plate regression spline smoothing term. Continuous explanatory variables were standardized to zero mean and divided by 2 standard deviations [66] to enable relative interpretation of averaged model coefficients. Models were ranked according to their Akaike Information Criterion (AIC) (Occurrence analysis), or QAIC (Prevalence analysis) for over-dispersed data, due to inflated residual deviance compared to degrees of freedom. Model averaging was then conducted across all models with *Δ*(Q)AIC < 6 [60]. All analyses were repeated for each ranavirosis criteria. Variables were classed as significant if the 95% confidence intervals did not span zero [61]. As an indication of the usefulness of any findings for real life application, the proportion of deviance explained by the models was noted (calculated as 1-residual deviance/null deviance).

## Results

**Fig 1:**
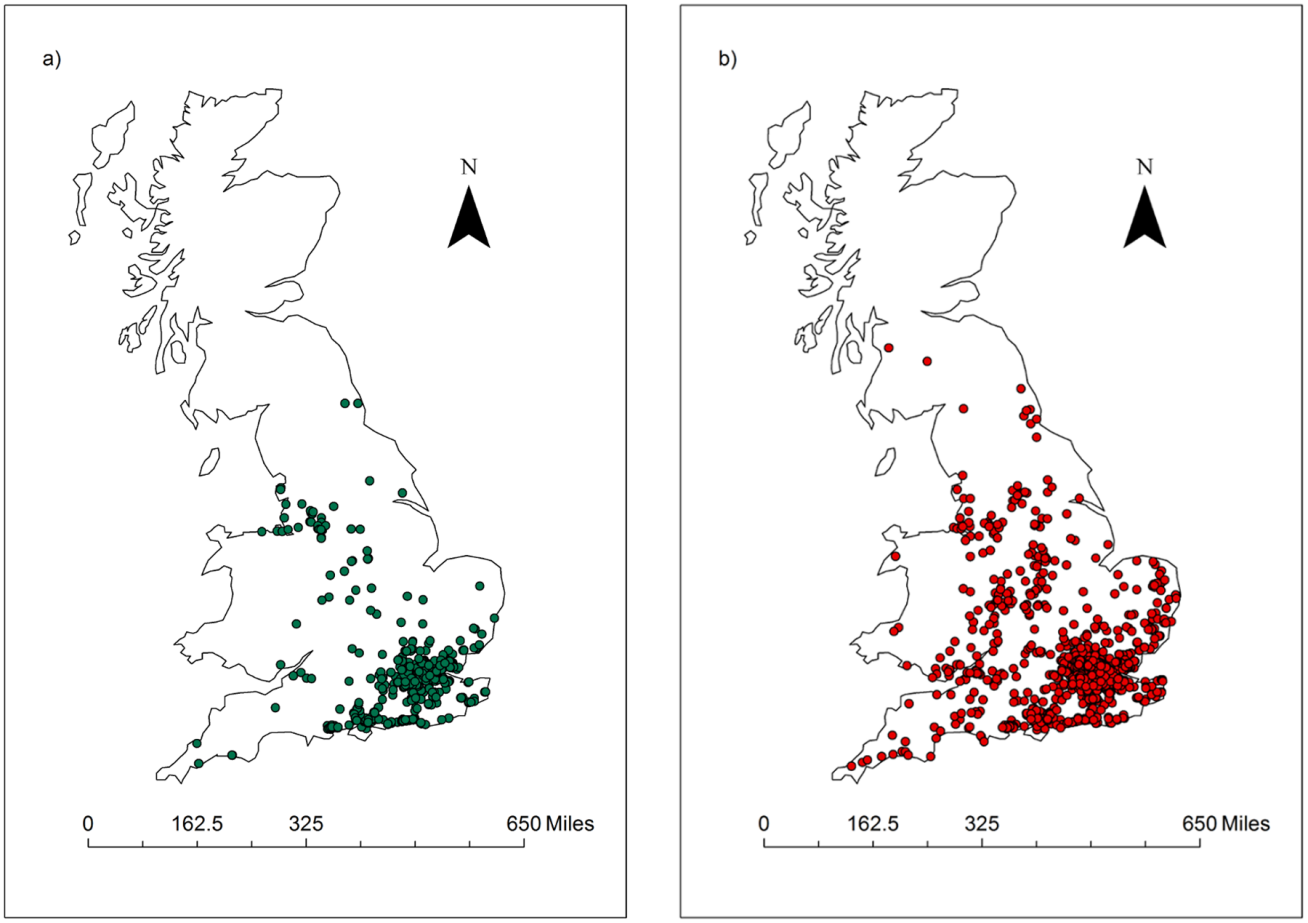
Spatial distribution of amphibian mortality records. Distribution of amphibian mortality records analysed here (1992–2000) showing a) Ranavirosis positive records and b) Ranavirosis negative records (Occurrence analysis Criteria 1; n = 2,113).

Using criteria 1 [9] for identifying *Ranavirus*-consistent mortalities, and excluding incomplete and duplicate records resulted in 702 *Ranavirus*-consistent records and a total of 2,113 records (Fig 1). The magnitude of a *Ranavirus*-consistent mortality event ranged between 1 and 251 individuals (Median: 15 individuals).

### a) Factors affecting ranavirosis occurrence

**Fig 2:**
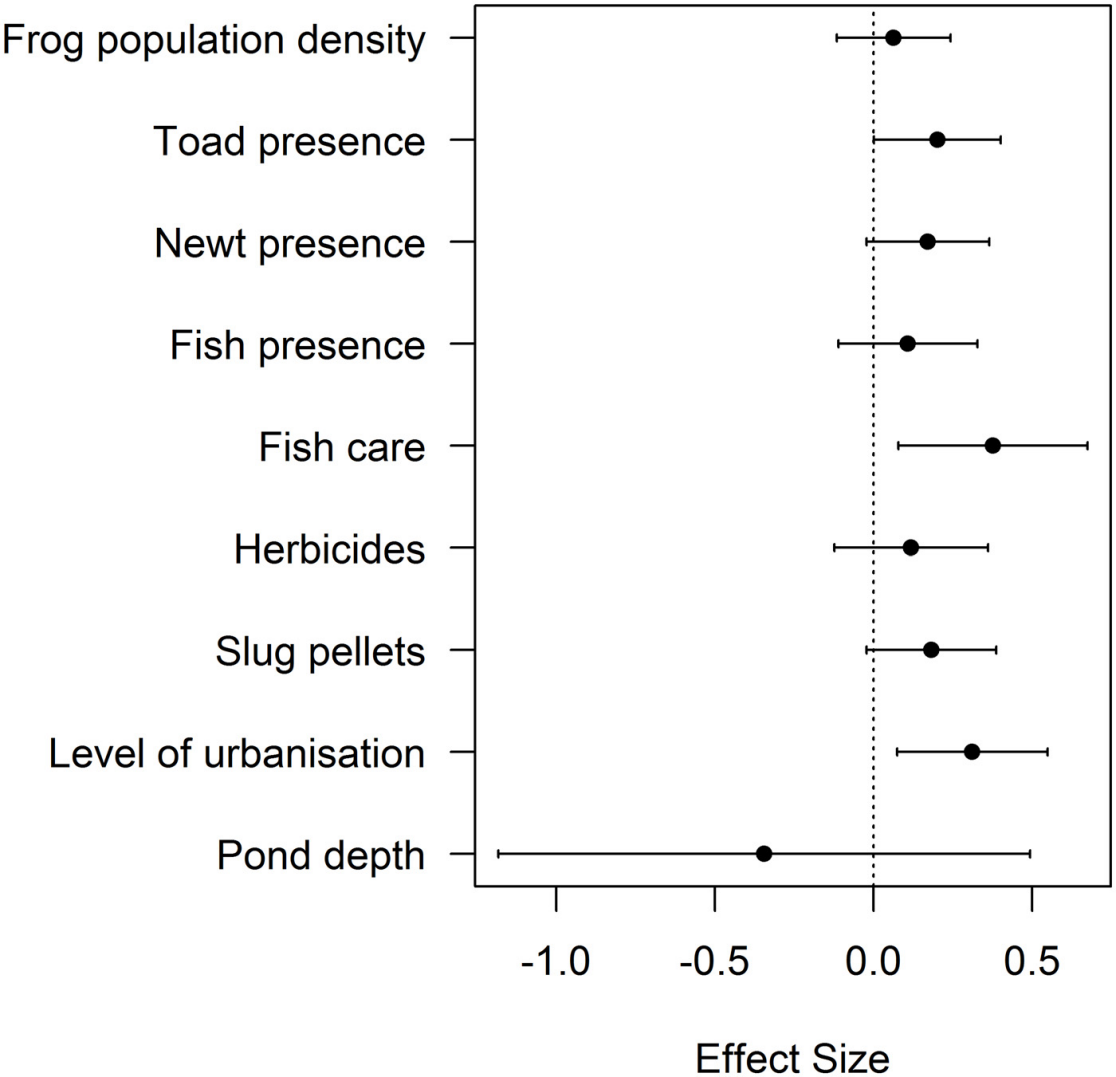
Abiotic and Biotic Variables Influencing Ranavirosis Occurrence. Model estimates and 95% confidence intervals for top ranking parameters Δ*AIC* <6 for ranavirosis occurrence (Criteria 1; [9]). Zero is indicated with a dotted line to demonstrate the importance of parameters in which confidence intervals do not overlap zero. Effect sizes above zero denote a positive relationship between each variable and ranavirosis occurrence.

Ranavirosis occurrence was best explained by the presence of toads (Confidence Intervals (CI): 0.002, 0.402), being in an urban environment (CI: 0.074, 0.549) and the use of fish care products (CI: 0.079, 0.675) (Fig 2, S2 Table). Confidence intervals of these parameters did not span zero and positive trends were found for each variable, with each increasing the likelihood of ranavirosis occurrence (Fig 2). All other variables had confidence intervals that spanned zero. As would be expected for disease cases, a geographic pattern of disease occurrence was detected (χ^2^_14.38_ = 55.67, p<0.0001). The inclusion of the northings and eastings of each mortality event therefore ensured independence of residuals for the variables of interest. The explanatory power of this model (Spatial model: 4.39% deviance explained, non-spatial model: 1.50% deviance explained), was low.

### b) Factors affecting ranavirosis prevalence

**Fig 3:**
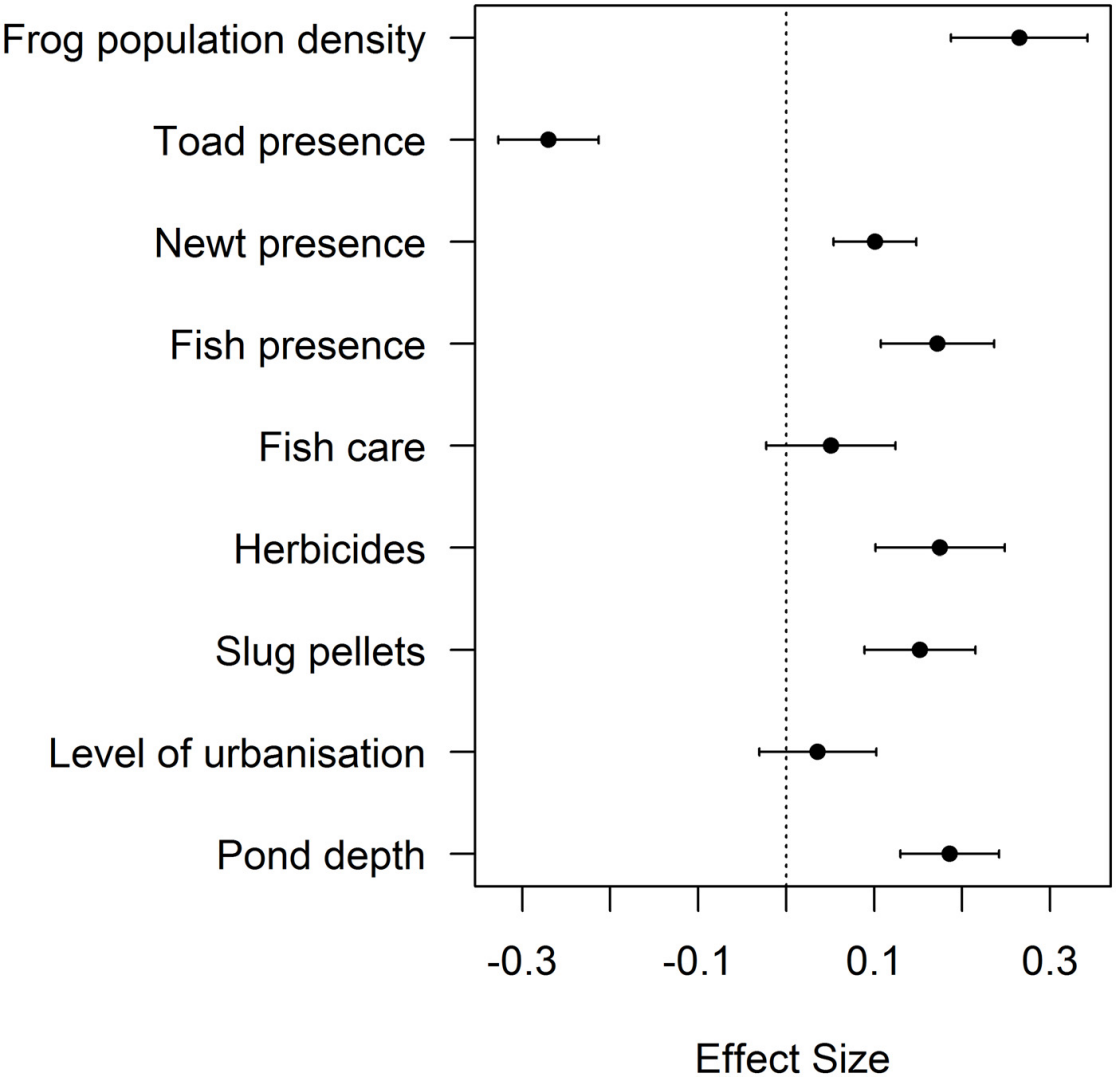
Abiotic and Biotic Variables Influencing Ranavirosis Prevalence. Model estimates and 95% confidence intervals for top ranking parameters *Δ* QAIC <6 for ranavirosis prevalence (Criteria 1; [9]). Zero is indicated with a dotted line to demonstrate the importance of parameters in which confidence intervals do not overlap zero. Effect sizes below zero indicate a negative relationship between the variable and ranavirosis prevalence and effect sizes above zero indicate a positive relationship.

A negative association was found between ranavirosis prevalence in common frogs and the presence of toads (CI: -0.327, -0.213), with a large effect size and confidence intervals not overlapping zero (Fig 3, S3 Table). In order of largest effect size first, ranavirosis prevalence was positively associated with frog population density (CI: 0.188, 0.343), pond depth (CI: 0.130, 0.242), the presence of fish (CI: 0.107, 0.236), the use of herbicides (CI: 0.101, 0.248) and slug pellets (CI: 0.089, 0.215), and the presence of newts (CI: 0.054, 0.148). The use of fish care products and the level of urbanisation did not help explain ranavirosis prevalence, with confidence intervals that spanned zero. The geographic distribution of mortality events was important in explaining ranavirosis prevalence (χ^2^_28.22_ = 850.4, p<0.001) and so its inclusion ensured independence of residuals for the variables of interest. The deviance explained by this model was 18.1% compared to the non-spatial equivalent of 5.5% deviance explained.

### Comparing ranavirosis criteria

When considering Criteria 2 [17], 740 reports were considered *Ranavirus*-consistent and an additional four variables helped explain ranavirosis occurrence: frog population density (CI: 0.042, 0.410), newt (CI: 0.116, 0.495) and fish presence (CI: 0.077, 0.515) and the use of slug pellets (CI: 0.055, 0.455) (See S4 Table). The deviance explained by this model was again very low at 5.07% but higher than the non-spatial equivalent (Deviance explained 2.7%). Ranavirosis prevalence results were very similar between criteria, though the level of urbanisation was important for Criteria 2 only (CI: -0.247, -0.133) and the direction of significance differed for pond depth (CI: -0.211, -0.118) (See S5 Table). The deviance explained by this model was 21.1%, which is marginally higher than found for criteria 1 and much higher than the non-spatial equivalent (Deviance explained 6.2%).

Due to the differences between the results using criteria 1 and 2, we re-ran the analysis excluding mortality events outside of May-September from criteria 2 [17], as this was the main difference between the criteria. This re-analysis resulted in 653 *Ranavirus*-consistent mortality events and both criteria subsequently having very similar predictors for ranavirosis occurrence and prevalence. There was no qualitative difference in results when excluding mortalities outside of May-September for the occurrence analysis (See S6 Table, Deviance explained 7.18%, deviance explained by the non-spatial model 3.33%). The exclusion of mortality events outside of May-September resulted in a positive association between ranavirosis prevalence and pond depth (CI: 0.048, 0.149) and no longer any effect of level of urbanisation (CI: -0.124, 0.005) (See S7 Table, Deviance explained 15.3%, Deviance explained of non-spatial model 5.5%).

## Discussion

This study has yielded insights into the factors associated with the occurrence and prevalence of ranavirosis in UK common frogs, highlighting the role of biotic *and* abiotic processes in its impacts. Many of these abiotic characteristics are anthropogenic, highlighting the potential role for human intervention to limit the impacts of this disease. When considering the first disease criteria (Criteria 1, [9]), increased ranavirosis prevalence was associated with increasing frog population density, the presence of fish and newts, increasing pond depths and the use of garden chemicals. Decreased prevalence was associated with the presence of toads (Fig 3, S3 Table). Ranavirosis occurrence was best explained by the presence of toads, an urban setting and the use of fish care products (Fig 2, S2 Table), but these predictors had low explanatory power. Spatial models explained substantially more deviance than non-spatial, highlighting the importance of accounting for the geographic distribution of mortality events.

### Biotic drivers

The strong association found here between fish presence and increased ranavirosis prevalence (using both criteria), but only weak evidence for an association between fish presence and the occurrence of ranavirosis (only apparent with criteria 2) may suggest that fish are not necessarily a direct source of infection. Common frogs are known to be susceptible to pike-perch iridovirus in experimental conditions [67], and in the wild fish may amplify environmental viral levels, increase the density of potential disease reservoirs, or influence immune function through energy trade-offs or stress hormone production induced from predation risk [68]. Fish susceptibility to *Ranavirus* appears to be low [69], with mortality being in the region of 0–10% [15], [69] but with the ability for viral replication and transfer of the virus to other vertebrate classes [15], [69]. Mosquito fish *Gambusia affinis* for example have been shown to transfer *Ranavirus* to 10% of conspecific tadpoles in a controlled environment whilst no fish mortality was detected when tadpoles were initially infected [15]. Whilst *Ranavirus* could not be re-isolated from experimentally infected goldfish [70], these were not infected with UK isolates and so the amplification ability of fish commonly stocked in ponds across the UK is currently unknown.

Alternatively or additionally, predator presence can result in reduced amphibian foraging rates and have subsequent knock on effects for immune system development [68], [71]. Fish are known predators of pre-metamorphic amphibians [72], [73] and wood frog tadpoles exhibited delayed development in the presence of predatory dragonfly larvae, resulting in reduced immune function [68]. Dragonfly predator cues have also been shown to decrease survival when in combination with *Ranavirus* exposure for larval tiger salamanders [34]. Further work is needed to confirm the impacts of early life predator exposure on adult common frog immunity. Future experiments should determine viral replication rates in commonly stocked pond fish, the effects fish predator cues have on immune function in common frogs throughout life history stages, and how these effects differ among different fish species.

*Ranavirus* is a known infector of whole communities [74], which could explain the associations found here with newt presence and increased ranavirosis prevalence, as well as toad presence and decreased ranavirosis prevalence. Whilst newt species present in the reported ponds was unknown, smooth newts have tested positive for *Ranavirus* infection in the UK [41], and alpine and palmate newts are known to be susceptible in other European countries [8], [10]. The fact that only one case of *Ranavirus* mortality of newts in the UK has been published [41] and in our study they were associated with increased prevalence but not occurrence, could make them amplifying hosts rather than sustaining the disease themselves. The association between toad presence and ranavirosis occurrence (supported only by criteria 1) and decreased prevalence (supported by all criteria) could suggest toads contribute to the spread of the disease, but may be less competent hosts, resulting in reduced transmission efficiency and a dilution effect in their presence [47]. Transmission efficiency between adult common frogs and common toads is currently unknown, though studies on other amphibian communities show differing *Ranavirus* transmission efficiencies between different species, and different severity outcomes depending on which species is infected first [75]. Experimental infection of common frog and toad tadpoles with the same *Ranavirus* strains showed less severe disease and infection rates in toads compared to frogs, suggesting lower susceptibility of toads to *Ranavirus* [40]. This is in contrast to recent findings that show wild common toad populations to be experiencing *Ranavirus*-consistent mass mortality and population declines in Spain, albeit due to a different viral lineage (Common midwife toad virus) [10]. Further experimental work is needed to determine the roles of both newts and toads in common frog disease prevalence, and to determine whether toad and newt density play an important role.

Increased population density of common frogs was an important determinant of increased ranavirosis prevalence, with a large effect size. Whilst this relationship is correlational, it is suggestive of density dependent transmission. This would suggest natural regulation of disease levels, with low transmission efficiency at low densities resulting in reduced pathogen persistence [76]. Further research with more accurate population density estimates are needed to confirm this pattern, especially since strong associations between fish and newt presence and ranavirosis prevalence have been found, which could allow *Ranavirus* persistence at low primary host densities. Frequency dependent transmission has been found in *Ranavirus*-amphibian systems in North America [53], and previous work in the UK found three outcomes after the first *Ranavirus-*caused die-off in common frog populations—population extinction, persistent infection or recovery [9]—which could indicate a combination of both frequency and density dependent transmission [77].

### Abiotic drivers

Deeper ponds were associated with increased ranavirosis prevalence. Pond characteristics may have important consequences for disease prevalence due to the short dispersal distances of common frogs [55], [56] and the pathogen’s ability to persist in both pond water and sediment [51]. Deeper ponds could be linked to a multitude of biotic and abiotic characteristics that could influence disease prevalence, such as differing temperatures [21], [78] associated with thermal stratification, water quality [36], levels of emergent vegetation [79] or the presence of predators [34].

The second criteria for *Ranavirus*-consistent mortalities confirmed the positive association between pond depth and disease prevalence, but only when mortalities outside of May-September were excluded from the analysis. Deeper ponds may therefore have protective qualities for overwintering frogs, providing decreased likelihood of freezing, cooler temperatures and higher oxygen levels [80], though dissolved oxygen content is likely influenced by multiple factors [81]. Whilst common frogs are thought to be predominantly aquatic hibernators [82], terrestrial hibernation does also occur [83]. Deeper ponds may also be correlated with larger ponds and larger gardens, which may provide more terrestrial hibernacula.

Ranavirosis occurrence was associated with more urban areas, and previous work suggests increased disease prevalence in relation to human modified landscapes is fairly common across taxonomic groups [84]. For example, increased chronic wasting disease prevalence in mule deer was associated with more developed land use [85] and West Nile Virus antibody prevalence was increased in urban compared to rural songbirds [86]. The mechanisms behind these patterns however differ greatly among host-pathogen systems. Here, the association between ranavirosis occurrence and urban areas may be due to anthropogenic barriers between populations reducing genetic variation and impacting on fitness [87] and disease susceptibility [19] or increased exchange of pond materials between gardens and increased likelihood of introduced species. Densely populated areas will likely be associated with international trade, which is a known route of *Ranavirus* spread [14], [26–28]. Indeed, urban areas are associated with introduced species [88] and species that have been introduced into the UK such as the alpine newt are susceptible to *Ranavirus* [8], [42]. It has also been speculated that introduced North American bullfrogs and goldfish have been involved in the spread of *Ranavirus* into the UK from its origin in North America [14], [26]. The dataset analysed here only contained records of the presence of ornamental fish, and the full extent of other non-native species in the ponds analysed was unknown.

Level of urbanisation did not influence ranavirosis prevalence according to criteria 1; [9], but rural areas were associated with increased prevalence according to criteria 2; [17]. This could be linked to agricultural run-off associated with rural landscapes and subsequent amphibian immunosuppression [32], [89]. However, when excluding mortalities outside of May-September, increased ranavirosis prevalence was no longer associated with rural areas, suggesting it is the mortalities in cooler months driving this pattern, perhaps due to harsher winter conditions in rural areas increasing general mortality. Urban and rural common frog populations are known to differ in growth rates [90], gene flow [87] and heavy metal levels [91] but it is unclear how these may interact with disease susceptibility and season to explain this association.

Chemicals used to manage gardens and ponds are known to influence amphibian immune function, with even low pesticide doses resulting in reduced antibody production in leopard frogs (*Rana pipiens*) [31]. Slug pellets and herbicides are pesticides well known for their detrimental impacts on wildlife [92–94] and here their use was associated with increased ranavirosis prevalence. Experimental studies on North American species corroborate these findings with increased susceptibility of tiger salamander (*Ambystoma tigrinum*) larvae to *Ranavirus* infection when exposed to the herbicide atrazine [32] and insecticide chlorpyrifos [33]. Alternatively, the association found with slug pellet use could be due to a correlated unrecorded variable rather than the presence of the chemicals themselves. For example, slug pellet use is likely linked to high slug activity, which in turn could be linked to increased temperatures [95] and viral replication, as *Ranavirus* virulence can be temperature dependent [78]. A better understanding of the associations found between pesticides and ranavirosis prevalence could be determined with further information on quantities of chemicals used, as increasing pesticide concentration has been associated with increased *Ranavirus* infection rates [33]. Propensity to use fish care products could be influenced by the general health and condition of the pond, which could explain the association found here between fish care use and ranavirosis occurrence, though this is unclear and requires further investigation.

The criteria used to define ranavirosis cases were robust, with similar outcomes across analyses. The slight differences between criteria in prevalence analyses were re-aligned by the exclusion of mortalities outside of May-September. Our conclusions require some caution because the data were generated by citizens and measurements of environmental variables could not be formally validated. However, the large sample sizes, and informal validation of a subset of data, give us confidence in the conclusions drawn. The explanatory power of the models likely reflect the complexity of wild disease systems which cannot be fully captured by the predominant use of binary variables. The largest predictor of ranavirosis occurrence may more likely be the history of *Ranavirus* in the area or other environmental conditions such as temperature, which could influence virus replication rate and subsequent detection [13]. Due to the citizen science nature of the data collection, and the fine scale differences in temperature that would be likely within and between gardens, temperature could not be included in the models.

Higher predictive power of prevalence could likely be achieved by considering population differences in immune defence, genetic variation and virulence of alternative viral genotypes. Directional selection of the Major Histocompatibility Complex in *Ranavirus* infected populations for improved immunity has been suggested [96], meaning prevalence may be influenced by how long the population has been subjected to *Ranavirus*, with the potential for a co-evolutionary arms race between *Ranavirus* and host. *Ranavirus* susceptibility has also been found to correlate with genetic diversity, with low diversity associated with increased mortality [19], which could make connectivity of populations another important predictor of risk. A more comprehensive survey of diversity among UK ranaviruses and a better understanding of the impact of virus genotype on virulence could also help explain differences in prevalence. Recent research shows amphibian mortality rates differ according to both the host and parasite genotype, with different *Ranavirus* isolates causing different mortality rates in different species and genotypes, but with temperature also influencing these relationships [97]. It is also important to note that the covariates considered here could have caused sub-lethal affects, such as impacts on growth rates [98] that are not detected in this study due to the focus on mortality.

### Conclusions and management implications

*Ranavirus* is a global issue [5], and has already been implicated in global mass mortalities and the declines of amphibian populations in Spain and the UK [5], [9], [10]. An increased understanding of the causes of spread of *Ranavirus* and prevalence of ranavirosis are vital in limiting the impacts of one of several threats—alongside habitat loss and fragmentation—facing amphibians in the UK [58]. We highlight the role of both ecological and anthropogenic processes as drivers of disease in common frogs. Whilst these should be interpreted with caution, this is the first time that fine scale biotic and abiotic characteristics have been associated with ranavirosis in free living populations in the UK. Though the effects of any garden management changes may be small, these findings represent large scale patterns and should therefore have useful applications for decreasing the number of deaths caused by *Ranavirus*. Beneficial management practises will include reducing the use of herbicides and slug pellets, especially in the summer months when *Ranavirus* mortalities are at their highest [23], alongside limiting the introduction of non-native species. Whilst our results suggest deeper ponds have the risk of increased ranavirosis prevalence, we do not recommend the in-fill of deep ponds until we understand the mechanistic link between pond depth and disease. There are an estimated 2.5–3.5 million ponds in the UK [99], many of which are stocked with ornamental fish, and public opinion surveys suggest garden chemical use is high (71% of respondents use at least one chemical product) [100]. Pond owners care about the health of their ponds, as evidenced by their mass participation in the Frog Mortality Project over the past twenty years. Furthermore, nearly half of respondents opposing the use of fertilizers in the countryside did so due to the damage caused to wildlife [100]. These factors demonstrate the potential for garden owners to decrease the impacts of human activities on ranavirosis prevalence and the health of garden wildlife as a whole.

## Supporting Information

S1 File Validation of total frog population size estimates. (DOCX)

S1 Table Parameters included in all global models. Parameters included in all global models and reasons for their potential relevance to ranavirosis occurrence and prevalence.
(DOCX)

S2 Table Abiotic and Biotic Variables Influencing Ranavirosis Occurrence for Criteria 1. Estimates, standard error and confidence intervals for factors affecting ranavirosis occurrence as defined by Criteria 1.
(DOCX)

S3 Table Abiotic and Biotic Variables Influencing Ranavirosis Prevalence for Criteria 1. Estimates, standard error and confidence intervals for factors affecting ranavirosis prevalence as defined by Criteria 1.
(DOCX)

S4 Table Abiotic and Biotic Variables Influencing Ranavirosis Occurrence for Criteria 2. Estimates, standard error and confidence intervals for factors affecting ranavirosis occurrence as defined by Criteria 2.
(DOCX)

S5 Table Abiotic and Biotic Variables Influencing Ranavirosis Prevalence for Criteria 2. Estimates, standard error and confidence intervals for factors affecting ranavirosis prevalence as defined by Criteria 2.
(DOCX)

S6 Table Abiotic and Biotic Variables Influencing Ranavirosis Occurrence for Criteria 2 Excluding Mortalities outside of May-September. Estimates, standard error and confidence intervals for factors affecting ranavirosis occurrence as defined by Criteria 2 but with mortalities outside of May-September excluded.
(DOCX)

S7 Table Abiotic and Biotic Variables Influencing Ranavirosis Prevalence for Criteria 2 Excluding Mortalities outside of May-September. Estimates, standard error and confidence intervals for factors affecting ranavirosis prevalence as defined by Criteria 2 but with mortalities outside of May-September excluded.
(DOCX)
