# Supplementary material for: Anthropogenic and Ecological Drivers of Amphibian Disease (Ranavirosis)
Source: PLoS One. 2015 Jun 3;10(6):e0127037. doi: 10.1371/journal.pone.0127037 (PMC4454639; doi:10.1371/journal.pone.0127037)
Supplement: S1 File — (DOCX) [file pone.0127037.s001.docx]

**S1 Supporting Information. Validation of total frog population size estimates**

Total population size as determined by pond owners was validated by conducting correlation analyses against two additional independent measures of population size recorded within the database (Spawn clump counts and square feet of spawn). Spawn clump counts are regularly used for estimating the number of adult females within a population, which can then be multiplied by two for a total population size estimate. The size of spawn clumps (in sq feet) is an additional method for population estimates, and has been shown to be strongly correlated with number of spawn clumps, and a more reliable method for inexperienced recorders [1]. After removal of potential duplicate records from the same postcode, all reports from the database containing complete information for the variables required for each analysis were included.

Population size as estimated by pond owners was found to be significantly correlated with the number of spawn clumps multiplied by two (Spearman rho correlation r_s_ = 0.371, p < 0.001, n=1,460). Considering only ponds containing spawn, the number of spawn clumps was significantly correlated with square foot of spawn (Spearman rho correlation, r_s_ = 0.605, p < 0.001, n=456) giving validity to the population estimates used within subsequent analyses.

**References**

1. Griffiths RA, Raper SJ. How many clumps in a mass of frogspawn? British Herpetological Society Bulletin. Br Herpetol Bull. 1994:50; 14–17.
