# Supplementary material for: Anthropogenic and Ecological Drivers of Amphibian Disease (Ranavirosis)
Source: PLoS One. 2015 Jun 3;10(6):e0127037. doi: 10.1371/journal.pone.0127037 (PMC4454639; doi:10.1371/journal.pone.0127037)
Supplement: S1 Table — Parameters included in all global models and reasons for their potential relevance to ranavirosis occurrence and prevalence. (DOCX) [file pone.0127037.s002.docx]

**S1 Table. Parameters included in all global models and reasons for their potential relevance to ranavirosis occurrence and prevalence**

| **Parameter** | **Defined as:** | ***A priori* justification for inclusion** | **Citations** |
| --- | --- | --- | --- |
| Frog density | Total frog population size (See S1 Supporting Information for validation) / Total pond volume (Depth x width x length) | Indication of density/frequency dependent transmission | [1]-[5] |
| Toad presence | Presence/Absence | Potential reservoir/host that could result in amplification/dilution | [6],[7] |
| Newt presence | Presence/Absence | Potential reservoir/host that could result in amplification/dilution | [7]-[9] |
| Fish presence | Presence/Absence | Potential reservoir/host that could result in amplification/dilution | [10]-[12] |
| Fish care | Used that year/Not used | Chemicals causing immunosuppression | [13] |
| Herbicides | Used/Not used | Chemicals causing immunosuppression | [13] |
| Slug pellets | Used/Not used | Chemicals causing immunosuppression | [13] |
| Level of urbanisation | Urban/Rural | Human activity/trade/introduced species | [14]-[16] |
| Pond depth | Average depth of garden ponds (m) | Temperature dependent effects | [17] |

**References**

1. Duffus ALJ. *Ranavirus* ecology in common frogs (*Rana temporaria*) from the United Kingdom: transmission dynamics, alternate hosts and host-strain interactions. PhD thesis, Queen Mary University of London and Zoological Society London; 2009.
2. Teacher AGF, Cunningham AA, Garner TWJ. Assessing the long-term impact of *Ranavirus* infection in wild common frog populations. Anim Conserv. 2010;13: 514-522.
3. Nazir J, Spengler M, Marschang RE. Environmental persistence of amphibian and reptilian ranaviruses. Dis Aquat Organ. 2012;98: 177-184.
4. Harp EM, Petranka JW. *Ranavirus* in wood frogs (*Rana sylvatica*): potential sources of transmission within and between ponds. J Wildlife Dis. 2006;42: 307-318.
5. Brunner JL, Schock DM, Collins JP. Transmission dynamics of the amphibian *Ranavirus* *Ambystoma tigrinum* virus. Dis Aquat Org. 2007;77: 87-95.
6. Cunningham AA, Hyatt AD, Bennett PM. Experimental transmission of a *Ranavirus* disease of common toads (*Bufo bufo*) to common frogs (*Rana temporaria*). Epidemiol Infect. 2007;135: 1213-1216.
7. Duffus ALJ. *Ranavirus* ecology in common frogs (*Rana temporaria*) from the United Kingdom: transmission dynamics, alternate hosts and host-strain interactions. PhD thesis, Queen Mary University of London and Zoological Society London; 2009.
8. Balseiro A, Dalton KP, Cerro AD, Marquez I, Parra F, Prieto JM, Casais R. Outbreak of common midwife toad virus in alpine newts (*Mesotriton alpestris cyreni*) and common midwife toads (*Alytes obstetricans*) in Northern Spain: A comparative pathological study of an emerging *Ranavirus*. Vet J. 2010;186: 256-258.
9. Price SJ, Garner TWJ, Nichols RA, Balloux F, Ayres C, Mora-Cabello de Alba A, et al. Collapse of amphibian communities due to an introduced *Ranavirus*. Curr Biol. 2014;24: 2586-2591.
10. Hyatt AD, Gould AR, Zupanovic Z, Cunningham AA, Hengstberger S, Whittington RJ, et al. Comparative studies of piscine and amphibian iridoviruses. Arch Virol. 2000;145: 301-331.
11. Cunningham AA, Daszak P, Rodriguez JP. Pathogen pollution: Defining a parasitological threat to biodiversity conservation. J Parasitol. 2003;89: S78-S83.
12. Mao J, Green DE, Fellers G, Chinchar VG. Molecular characterization of iridoviruses isolated from sympatric amphibians and fish. Virus Res. 1999;63: 45-52.
13. Albert A, Drouillard K, Haffner GD, Dixon B. Dietary exposure to low pesticide doses causes long-term immunosuppression in the leopard frog (*Rana pipiens*). Environ Toxicol Chem. 2007;26: 1179-1185.
14. St-Amour V, Wong WM, Lesbarrères D. Anthropogenic Influence on prevalence of 2 amphibian pathogens. Emerg Infect Dis. 2008;14: 1175-1176.
15. Schloegel LM, Picco AM, Kilpatricka AM, Davies AJ, Hyatt AD, Daszak P. Magnitude of the US trade in amphibians and presence of *Batrachochytrium dendrobatidis* and *Ranavirus* infection in imported North American bullfrogs (*Rana catesbeiana*). Biol Conserv. 2009;142: 1420-1426.
16. Riley SPD, Busteed GT, Kats LB, Vandergon TL, Lee LFS, Dagit RG, et al. Effects of urbanization on the distribution and abundance of amphibians and invasive species in southern California streams. Conserv Biol. 2005;19: 1894-1907.
17. Brand M, Gray M, Wilkes B, Brenes R, Miller D. Water temperature affects susceptibility to *Ranavirus*. 2013 International Symposium on Ranaviruses, Tennessee; 2013.
