# Supplementary material for: Anthropogenic and Ecological Drivers of Amphibian Disease (Ranavirosis)
Source: PLoS One. 2015 Jun 3;10(6):e0127037. doi: 10.1371/journal.pone.0127037 (PMC4454639; doi:10.1371/journal.pone.0127037)
Supplement: S2 Table — Estimates, standard error and confidence intervals for factors affecting ranavirosis occurrence as defined by Criteria 1. (DOCX) [file pone.0127037.s003.docx]

**S2 Table. Abiotic and Biotic Variables Influencing Ranavirosis Occurrence for Criteria 1.** Estimates, unconditional standard error and confidence intervals of each parameter from model averaging of the top ranking models (*Δ* <6) for ranavirosis occurrence for criteria 1 [1]. Parameters with confidence intervals that do not span zero help explain ranavirosis occurrence (bolded). Spatial position of the mortality event significantly contributed to model fit (χ^2^ _14.38_ = 55.67, p<0.0001) and deviance explained was 4.39%, n=2,113.

| **Parameter** | **Estimate** | **Unconditional SE** | **Confidence Interval 2.5%** | **Confidence Interval 97.5%** |
| --- | --- | --- | --- | --- |
| Intercept | -1.248 | 0.151 | -1.545 | -0.952 |
| Frog density | 0.064 | 0.092 | -0.116 | 0.243 |
| **Toad presence** | **0.202** | **0.102** | **0.002** | **0.402** |
| Newt presence | 0.172 | 0.099 | -0.022 | 0.365 |
| Fish presence | 0.109 | 0.112 | -0.110 | 0.328 |
| **Fish care** | **0.377** | **0.152** | **0.079** | **0.675** |
| Herbicides | 0.119 | 0.124 | -0.124 | 0.361 |
| Slug pellets | 0.183 | 0.105 | -0.022 | 0.388 |
| **Level of urbanisation** | **0.312** | **0.121** | **0.074** | **0.549** |
| Pond depth | -0.344 | 0.427 | -1.182 | 0.494 |

**References**

1. Teacher AGF, Cunningham AA, Garner TWJ. Assessing the long-term impact of *Ranavirus* infection in wild common frog populations. Anim Conserv. 2010;13: 514-522.
