# Supplementary material for: Anthropogenic and Ecological Drivers of Amphibian Disease (Ranavirosis)
Source: PLoS One. 2015 Jun 3;10(6):e0127037. doi: 10.1371/journal.pone.0127037 (PMC4454639; doi:10.1371/journal.pone.0127037)
Supplement: S3 Table — Estimates, standard error and confidence intervals for factors affecting ranavirosis prevalence as defined by Criteria 1. (DOCX) [file pone.0127037.s004.docx]

**S3 Table. Abiotic and Biotic Variables Influencing Ranavirosis Prevalence for Criteria 1.** Estimates, unconditional standard error and confidence intervals for each parameter from model averaging of the top ranking models (Δ <6) for ranavirosis prevalence for criteria 1 [1]. Parameters with confidence intervals that do not span zero help explain ranavirosis prevalence (bolded). Negative estimates indicate reduced prevalence and positive estimates indicate increased prevalence.

| **Parameter** | **Estimate** | **Unconditional SE** | **Confidence Interval 2.5%** | **Confidence Interval**  **97.5%** |
| --- | --- | --- | --- | --- |
| Intercept | -0.309 | 0.071 | -0.448 | -0.170 |
| **Frog density** | **0.265** | **0.040** | **0.188** | **0.343** |
| **Toad presence** | **-0.270** | **0.030** | **-0.327** | **-0.213** |
| **Newt presence** | **0.101** | **0.024** | **0.054** | **0.148** |
| **Fish presence** | **0.172** | **0.033** | **0.107** | **0.236** |
| Fish care products | 0.051 | 0.037 | -0.023 | 0.124 |
| **Herbicides** | **0.175** | **0.038** | **0.101** | **0.248** |
| **Slug pellets** | **0.152** | **0.032** | **0.089** | **0.215** |
| **Level of urbanisation** | 0.036 | 0.034 | -0.031 | 0.102 |
| **Pond depth** | **0.186** | **0.029** | **0.130** | **0.242** |

**References**

1. Teacher AGF, Cunningham AA, Garner TWJ. Assessing the long-term impact of *Ranavirus* infection in wild common frog populations. Anim Conserv. 2010;13: 514-522.
