# Supplementary material for: Anthropogenic and Ecological Drivers of Amphibian Disease (Ranavirosis)
Source: PLoS One. 2015 Jun 3;10(6):e0127037. doi: 10.1371/journal.pone.0127037 (PMC4454639; doi:10.1371/journal.pone.0127037)
Supplement: S4 Table — Estimates, standard error and confidence intervals for factors affecting ranavirosis occurrence as defined by Criteria 2. (DOCX) [file pone.0127037.s005.docx]

**S4 Table. Abiotic and Biotic Variables Influencing Ranavirosis Occurrence for Criteria 2.** Estimates, unconditional standard error and confidence intervals for each parameter from model averaging of the top ranking models (Δ <6) for ranavirosis occurrence for criteria 2 [1]. Parameters with confidence intervals that do not span zero help explain ranavirosis occurrence (bolded). Spatial position of the mortality event significantly contributed to model fit (χ^2^ _16.5_=49.03 p<0.001) and deviance explained was low (5.07%), n=2,219.

| **Parameter** | **Estimate** | **Unconditional SE** | **Confidence Interval 2.5%** | **Confidence Interval**  **97.5%** |
| --- | --- | --- | --- | --- |
| **Intercept** | **-1.638** | **0.154** | **-1.940** | **-1.337** |
| **Frog density** | **0.226** | **0.094** | **0.042** | **0.410** |
| Toad presence | 0.149 | 0.100 | -0.047 | 0.344 |
| **Newt presence** | **0.306** | **0.097** | **0.116** | **0.495** |
| **Fish presence** | **0.296** | **0.112** | **0.077** | **0.515** |
| **Fish care products** | **0.410** | **0.151** | **0.114** | **0.707** |
| Herbicide | -0.043 | 0.123 | -0.285 | 0.198 |
| **Slug pellets** | **0.255** | **0.102** | **0.055** | **0.455** |
| **Level of urbanisation** | **0.457** | **0.121** | **0.221** | **0.694** |
| Pond depth | -0.077 | 0.151 | -0.373 | 0.219 |

**References**

1. Price SJ. Emergence of a virulent wildlife disease: using spatial epidemiology and phylogenetic methods to reconstruct the spread of amphibian viruses. PhD Thesis, Queen Mary University of London. 2013.
