# Supplementary material for: Anthropogenic and Ecological Drivers of Amphibian Disease (Ranavirosis)
Source: PLoS One. 2015 Jun 3;10(6):e0127037. doi: 10.1371/journal.pone.0127037 (PMC4454639; doi:10.1371/journal.pone.0127037)
Supplement: S5 Table — Estimates, standard error and confidence intervals for factors affecting ranavirosis prevalence as defined by Criteria 2. (DOCX) [file pone.0127037.s006.docx]

**S5 Table. Abiotic and Biotic Variables Influencing Ranavirosis Prevalence for Criteria 2.** Estimates, unconditional standard error and confidence intervals for each parameter from model averaging of the top ranking models (Δ <6) for ranavirosis prevalence for criteria 2 [1]. Parameters with confidence intervals that do not span zero help explain ranavirosis prevalence (bolded). Negative estimates indicate a negative association between the variable and ranavirosis prevalence and positive estimates indicate a positive association between the variable and ranavirosis prevalence. Spatial position of the mortality event significantly contributed to the model fit (χ^2^ _28.29_=1316, p<0.001). Deviance explained by the model was 21.1%, n= 740.

| **Parameter** | **Estimate** | **Unconditional SE** | **Confidence Interval 2.5%** | **Confidence Interval**  **97.5%** |
| --- | --- | --- | --- | --- |
| Intercept | -0.122 | 0.106 | -0.330 | 0.086 |
| **Frog density** | **0.186** | **0.022** | **0.143** | **0.229** |
| **Toad presence** | **-0.301** | **0.027** | **-0.353** | **-0.249** |
| **Newt presence** | **0.167** | **0.022** | **0.124** | **0.210** |
| **Fish presence** | **0.161** | **0.028** | **0.107** | **0.215** |
| Fish care | 0.062 | 0.034 | -0.005 | 0.130 |
| **Herbicide** | **0.163** | **0.028** | **0.107** | **0.218** |
| **Slug pellets** | **0.062** | **0.029** | **0.005** | **0.120** |
| **Level of urbanisation** | **-0.190** | **0.029** | **-0.247** | **-0.133** |
| **Pond depth** | **-0.165** | **0.024** | **-0.211** | **-0.118** |

**References**

1. Price SJ. Emergence of a virulent wildlife disease: using spatial epidemiology and phylogenetic methods to reconstruct the spread of amphibian viruses. PhD Thesis, Queen Mary University of London. 2013.
