# Supplementary material for: Anthropogenic and Ecological Drivers of Amphibian Disease (Ranavirosis)
Source: PLoS One. 2015 Jun 3;10(6):e0127037. doi: 10.1371/journal.pone.0127037 (PMC4454639; doi:10.1371/journal.pone.0127037)
Supplement: S6 Table — Estimates, standard error and confidence intervals for factors affecting ranavirosis occurrence as defined by Criteria 2 but with mortalities outside of May-September excluded. (DOCX) [file pone.0127037.s007.docx]

**S6 Table**. **Abiotic and Biotic Variables Influencing Ranavirosis Occurrence for Criteria 2 Excluding Winter Mortalities.**  Estimates, unconditional standard error and confidence intervals for each parameter from model averaging of the top ranking models (Δ <6) for ranavirosis occurrence for criteria 2 [1] but with winter mortalities excluded as per criteria 1 [2]. Parameters with confidence intervals that do not span zero help explain ranavirosis occurrence (bolded). Spatial position of the mortality event significantly contributed to model fit (χ^2^ _17.21_= 72.2, p<0.001) and deviance explained was 7.18%, n= 2,160.

| **Parameter** | **Estimate** | **Unconditional SE** | **Confidence Interval 2.5%** | **Confidence Interval 97.5%** |
| --- | --- | --- | --- | --- |
| Intercept | -1.923 | 0.163 | -2.243 | -1.604 |
| **Frog density** | **0.233** | **0.095** | **0.048** | **0.418** |
| Toad presence | 0.140 | 0.105 | -0.066 | 0.346 |
| **Newt presence** | **0.306** | **0.102** | **0.106** | **0.505** |
| **Fish presence** | **0.371** | **0.118** | **0.140** | **0.603** |
| **Fish care** | **0.492** | **0.154** | **0.189** | **0.795** |
| Herbicides | 0.028 | 0.130 | -0.227 | 0.282 |
| **Slug pellets** | **0.210** | **0.107** | **0.001** | **0.420** |
| **Level of urbanisation** | **0.528** | **0.130** | **0.273** | **0.782** |
| Pond depth | -0.125 | 0.233 | -0.581 | 0.332 |

**References**

1. Price SJ. Emergence of a virulent wildlife disease: using spatial epidemiology and phylogenetic methods to reconstruct the spread of amphibian viruses. PhD Thesis, Queen Mary University of London. 2013.
2. Teacher AGF, Cunningham AA, Garner TWJ. Assessing the long-term impact of *Ranavirus* infection in wild common frog populations. Anim Conserv. 2010;13: 514-522.
