# Supplementary material for: Anthropogenic and Ecological Drivers of Amphibian Disease (Ranavirosis)
Source: PLoS One. 2015 Jun 3;10(6):e0127037. doi: 10.1371/journal.pone.0127037 (PMC4454639; doi:10.1371/journal.pone.0127037)
Supplement: S7 Table — Estimates, standard error and confidence intervals for factors affecting ranavirosis prevalence as defined by Criteria 2 but with mortalities outside of May-September excluded. (DOCX) [file pone.0127037.s008.docx]

**S7 Table. Abiotic and Biotic Variables Influencing Ranavirosis Prevalence for Criteria 2 Excluding Winter Mortalities**. Estimates, unconditional standard error and confidence intervals for each parameter from model averaging of the top ranking models (Δ <6) for ranavirosis prevalence for criteria 2 [1] but with winter mortalities excluded, as per criteria 1 [2]. Parameters with confidence intervals that do not span zero help explain ranavirosis prevalence (bolded). Negative estimates indicate a negative association between the variable and ranavirosis prevalence and positive estimates indicate a positive association between the variable and ranavirosis prevalence. Spatial position of the mortality event significantly contributed to the model fit (χ^2^ _27.18_=679, p<0.001). Deviance explained by the model was 15.3%, n=653.

| **Parameter** | **Estimate** | **Unconditional SE** | **Confidence Interval 2.5%** | **Confidence Interval**  **97.5%** |
| --- | --- | --- | --- | --- |
| Intercept | -0.105 | 0.076 | -0.254 | 0.044 |
| **Frog density** | **0.224** | **0.024** | **0.178** | **0.270** |
| **Toad presence** | **-0.242** | **0.028** | **-0.296** | **-0.188** |
| **Newt presence** | **0.100** | **0.023** | **0.054** | **0.146** |
| **Fish presence** | **0.153** | **0.031** | **0.092** | **0.214** |
| Fish care | -0.017 | 0.036 | -0.088 | 0.055 |
| **Herbicide** | **0.155** | **0.032** | **0.092** | **0.219** |
| **Slug pellets** | **0.091** | **0.030** | **0.033** | **0.150** |
| Level of urbanisation | -0.060 | 0.033 | -0.124 | 0.005 |
| **Pond depth** | **0.098** | **0.026** | **0.048** | **0.149** |

References

1. Price SJ. Emergence of a virulent wildlife disease: using spatial epidemiology and phylogenetic methods to reconstruct the spread of amphibian viruses. PhD Thesis, Queen Mary University of London. 2013.
2. Teacher AGF, Cunningham AA, Garner TWJ. Assessing the long-term impact of *Ranavirus* infection in wild common frog populations. Anim Conserv. 2010;13: 514-522.
